# Emergency department utilization by people living with HIV released from jail in the US South

**DOI:** 10.1186/s40352-020-00118-2

**Published:** 2020-06-27

**Authors:** Alfredo G. Puing, Xilong Li, Josiah Rich, Ank E. Nijhawan

**Affiliations:** 1 Stanford University Medical Center, Palo Alto, CA USA; 2 Department of Clinical Sciences, University of Texas Southwestern Medical Center, Dallas, TX USA; 3 Department of Internal Medicine and Epidemiology, Warren Alpert Medical School of Brown University, Providence, RI USA; 4 Department of Internal Medicine, Division of Infectious Diseases, University of Texas Southwestern Medical Center, 5323 Harry Hines Blvd., Dallas, TX 75390-9169 USA; 5 Parkland Health and Hospital Systems, Dallas, TX USA

**Keywords:** Emergency department, HIV, Jail

## Abstract

**Background:**

Incarceration is disruptive to HIV care, often resulting in poor retention in care for people living with HIV (PLWH) after jail release. This gap in HIV care might result in potentially preventable emergency department (ED) utilization. We analyzed demographic, incarceration, socioeconomic and clinical data for PLWH released from the Dallas County Jail to the community (1450 incarcerations, 1155 unique individuals) between January 2011 and November 2013.

**Results:**

The study population consisted of predominantly men (77%), with a mean age of 39 years, 67% were black and 14% were Hispanic; half of the releasees visited the ED at least once during the first-year post-jail. In adjusted analyses, female gender, family awareness of HIV status, serious mental illness, and late engagement to HIV care were significantly associated with higher ED utilization. Compared to the general Dallas population, PLWH released from jail had a 5-fold higher proportion of ED visits classified as related to substance use or mental health.

**Conclusions:**

Further efforts are needed to improve the transition from incarceration to community-based HIV care, substance use disorder treatment and mental health services, and to directly address re-engagement in HIV care for out-of-care PLWH who visit the ED.

## Introduction

Approximately 6.9 million adults in the US were under correctional supervision in 2013 (Glaze & Herberman, 2013). HIV/AIDS disproportionally affects the incarcerated population with rates up to three times higher than the general population (Bronson, 2017). In 2015, the HIV seroprevalence in state and federal prisons was 1.3% (Bronson, 2017), whereas in large urban jails, it was estimated to be between 1 to 6% (Beckwith et al., 2012; Spaulding et al., 2015). During incarceration, antiretroviral therapy (ART) is for the most part readily available and many detainees achieve an undetectable viral load prior to release (Iroh, Mayo, & Nijhawan, 2015). Nonetheless, PLWH face multiple barriers after transitioning from jail to their local communities which limit their re-engagement in HIV care (Loeliger et al., 2018; Westergaard, Spaulding, & Flanigan, 2013). For instance, it is estimated that only 36% of HIV-positive released detainees will present to an HIV provider within 6 months after release from jail or prison (Iroh et al., 2015).

This gap in HIV care may lead to increased utilization of acute care services, such as the emergency department (ED), in order to address needs like continuation of ART and treatment of non-emergent conditions. In a study evaluating ED utilization by individuals released from prison in Rhode Island, recently released persons presented to the ED mainly for evaluation and management of mental health disorders, substance use disorders, and ambulatory care conditions (Frank et al., 2013). Similarly, Meyer et al. examined ED utilization data from a cohort of HIV-positive individuals on ART released from prison and demonstrated that 1 out of 3 ED encounters were due to substance use, mental illness and/or social access issues (Meyer, Qiu, Chen, Larkin, & Altice, 2013). Frequent and early ED visits in HIV-positive jail releasees have been linked to poor socioeconomic support (i.e. homelessness) and substance use (Boyd, Song, Meyer, & Altice, 2015).

Although previous research studies on ED utilization following release from the criminal justice system have been informative, they have relied on the infrastructure of randomized controlled trials which provided increased oversight, readily available healthcare access, and scheduled discharge planning from prison to the community. Therefore, these studies may not reflect real-life conditions for most individuals released from jail. Another shortcoming of these prior studies is the limited healthcare network used to analyze the ED utilization among PLWH released from jail which likely misses a not insignificant number of outside-network ED encounters. Lastly, prior studies relied on small sample sizes that may not be representative of the entire population, and furthermore, could have be underpowered to detect different patterns of ED utilization among subgroups (Boyd et al., 2015; Khanna, Leah, Fung, Antoniou, & Kouyoumdjian, 2019; Meyer et al., 2013).

We utilized a retrospective cohort study to provide a comprehensive assessment of ED utilization by matching data from HIV-positive released jail detainees (accounting for 1450 releases) with an extensive database of 90 hospitals in North Texas as well as with community-based HIV clinic electronic data sources. Additionally, we characterized these ED encounters using the New York University (NYU) ED visit severity algorithm, a novel method to classify ED encounters including the identification of potentially avoidable visits (Ballard et al., 2010). The aims of our study were to (1) determine the prevalence, timing and predictors of ED utilization by HIV-positive individuals up to 12 months after release; (2) describe and compare the ED utilization between those who engaged and did not engage in HIV care after jail release; and (3) describe the primary reasons for ED visits, and whether or not they were potentially preventable.

## Methods

### Study population

This retrospective cohort study included all incarcerations among individuals with HIV at the Dallas County Jail (DCJ) that resulted in release to the community between January 1, 2011 and November 30, 2013. An incarceration was excluded from our sample if it did not result in a community release (e.g., transferred to prison or state jail) or if the inmate reported they planned to receive care with a provider outside of the Dallas Ryan White-funded clinic system. We included only Ryan White funded patients because the vast majority (93% in our study) of patients who come through jail qualify for Ryan White services and receive HIV services form a Ryan White funded clinic. In addition, due to the Health Resources and Services Administration requirement for annual reporting on all clients using the encrypted unique client identifier (eUCI), we were able to systematically capture clinic visit data through the Ryan White Services report which would otherwise not be readily available. Post-release periods were excluded if they were < 90 days before the end of the study period as per our previous approach to evaluate time to linkage to HIV care (Ammon et al., 2018). ED visits were tracked for 12 months following release from jail and including data through November 30, 2014, which marks the end of the study period. This study was approved by The University of Texas Southwestern Institutional Review Board.

In the DCJ, individuals are identified as HIV-positive if they disclose their HIV status during medical intake, if they are known to be HIV-positive from prior incarcerations, or if they have a positive HIV test result during incarceration. There are approximately 125 HIV-positive people (2% of population) in DCJ at any point in time, and HIV providers from Parkland Health and Hospital System (PHHS) are available 4 to 5 days per week. Of note, the majority of HIV-positive persons in DCJ are already known to have HIV at the time of entry (though date of diagnosis not routinely collected) with few new diagnoses given limited HIV testing during incarceration. During the study period, HIV discharge planning included an in-person case manager meeting while in jail, 1-week supply of medications provided upon request at release and a follow-up phone call from the case manager. In the Dallas metropolitan area, there is a large Ryan White-funded clinic system, including that operated by PHHS (providing care to over 5000 clinic patients per year during study period), and Prism Health (around 1500 patients per year during study period). The patient population that attends these clinics is predominately minority (approximately 50% black, 25% Hispanic), around 70% male, and nearly 50% of the overall outpatient clinic population are men who have sex with men.

### Data sources

A master data set was previously compiled from multiple sources, including the DCJ electronic medical record (Pearl, Business Computer Application), DCJ release data (Adult Information System), PHHS electronic medical record (Epic, Epic Systems Corporation), Prism Health clinic electronic medical record (Centricity, General Electronic Company), and Ryan White Service Report (RSR, used by HIV clinics for reporting to Health Resources and Services Administration). Initial matching of data was performed using an eUCI (described elsewhere) (Montague et al., 2016), with validation thorough manual review of jail and community electronic health records. Approximately 30% of the sample was not initially identified by eUCI matching alone, though all charts were manually reviewed for validation and additional data collection. Additional HIV-positive individuals, who were not matched using the eUCI method, were identified if “HIV” or “AIDS” was in the jail electronic health record problem list and validated through electronic chart review.

### Independent variables/predictors

Demographic variables include age, race/ethnicity, and gender. Age (as of November 30, 2013) was calculated using date of birth from the jail medical record. Race was collected from the RSR and ethnicity from the jail and outpatient clinic datasets, with race/ethnicity re-defined as non-Hispanic black, non-Hispanic white, or Hispanic. Gender was originally determined from the RSR and jail health record as female, male, or transgender. There were 14 incarcerations identified among transgender patients (all male to female), though due to this small size, gender at birth was used for the purposes of data analysis. HIV risk factor was extracted from the RSR, and narrowed down to history of injection drug use (IDU), men who have sex with men (MSM), or heterosexual/other. Individuals reporting both IDU and MSM were classified as IDU; those reporting both MSM and heterosexual risk were classified as MSM.

Marital status (ever married vs. other), family aware of HIV status (yes/no), and family supportive (yes/no) were obtained from jail case management documentation in Epic. Housing status was abstracted from the RSR and case management notes as a binary variable-stable/permanent vs. other. Whether someone was employed, unemployed, or on disability and self-reported history of sexual or physical abuse (yes/no) was obtained from case management notes. Alcohol use was condensed to a binary variable denoting at-risk alcohol use, defined as ≥14 drinks per week for men and ≥ 7 drinks per week for women, or not. A binary composite variable, “stimulant/opioid use,” was created for any reported use of cocaine, crack cocaine, methamphetamines, opioids and/or heroin. We created this combined variable due to our experience that these substances are associated with difficulty linking to care, and to separate them out from marijuana use, which is very prevalent in this population. Mental illness was collected as a binary composite variable with “serious mental illness” defined as having a diagnosis of bipolar disorder, psychosis, and/or schizophrenia. Self-reported information on prior use of an HIV clinic, prior prescription of ART, and prior adherence to ART before incarceration was available from case management and electronic medical record (EMR) data.

Income level was abstracted from the RSR as an ordinal variable based on percentage of federal poverty level (%FPL) using the earliest occurrence in the dataset for each individual. Health insurance coverage was collected from Epic and categorized as commercial insurance, Medicaid, Medicare, “charity” insurance (including Ryan White and local programs), or missing. Both income level and insurance coverage had significant amounts of missing data, and, since they were collected in community outpatient settings, the missing categories are collinear with not liking to care. Additionally, the income variable had a very limited range of responses (i.e., virtually every subject was under FPL). Due to these limitations, income and insurance were not included in the final analysis. Linkage to HIV care (yes/no) was defined as attending an ambulatory care visit with a Ryan White-funded HIV provider in Dallas County after release, as documented in the RSR and/or provider EMRs. If there was no documentation of such a visit, it was counted as failure to link to care for that incarceration. Though it is possible that individuals were engaged in care prior to incarceration and have a brief enough incarceration to still meet Ryan White criteria for retention in care (2 visits within 12 months > 90 days apart), we examined linkage to care at before and after 90 days after jail release given high baseline rates of ART non-adherence and post-release treatment interruption. In fact, given the disruptive impact of jail on a patient’s health, we generally recommend a visit within 30 days of release for all incarcerated PLWH.

### Outcomes

The primary outcome was post-release ED utilization, defined as frequency of ED visits within the first 12 months after jail release. This was categorized as no ED visit, 1 to 2 ED visits, and more than 3 ED visits to one or more of the 90 local hospitals that form part of the Dallas-Fort Worth Hospital Council Foundation (DFWHC). We chose to categorize ED visits rather than use them as a continuous variable since data would be skewed by some very heavy ED utilizers. We selected these three categories based on the literature (Frank et al., 2013) and the distribution of our data*.* Additionally, timing of first ED visit by linkage to HIV care, and NYU ED visit classification were described in order to further characterize ED utilization. We plotted daily cumulative percentage of having an ED visit or HIV clinic visit for the time frame post-release to first HIV clinic visit and first ED visit, separately, with censoring at the time of reincarceration or at the end of the study period. Linkage to HIV care was also classified in 3 categories: early linkage to HIV care (first HIV clinic visit before 90 days from jail release), late linkage to HIV care (first HIV clinic visit after 90 days from jail release), and no linkage to HIV care (no HIV clinic visit during the follow up period). The NYU ED visit severity algorithm classified the encounters into three main categories: emergent, non-emergent, and other (mental health related, alcohol related, substance abuse related, injury and unclassified). Emergent visits are further divided into three additional categories of preventability: ED care needed not preventable, ED care needed preventable, and primary care treatable (Ballard et al., 2010). ED utilization patterns among PLWH were compared to those of the general Dallas County population from 2010 to 2012 published by the DFWHC (Sharma, Seals, Jenkins, Isaacs, & Anderson, 2017).

### Statistical analyses

The main unit of analysis was each incarceration during the study period. Cross-tabular frequencies were used to calculate baseline characteristics. Univariate analyses were conducted, and associations between each independent variable and ED utilization as a categorical outcome (no ED visit, 1–2 ED visits, > 3 ED visits) were presented by an odds ratio with Wald confidence intervals. Subsequently, a multivariate ordinal logistic regression model was built by including all variables with a priori variables of age, gender, and length of incarceration included. The final model was constructing using a backward elimination selection method. Variables were retained in the model if *p* < 0.20. Multicollinearity was identified between the variables serious mental illness, stimulant/opioid use, and employment status, therefore employment was removed from the final multivariate model. In addition, though we present only the results of the multivariate model using incarceration as the unit of analysis, we also used a generalized linear model (GEE) to account for intra-subject correlation. Given similar results, this model is not presented in the results section.

### Missing data

Given the availability of comprehensive data sets from the jail, Ryan White funded clinics and DFWHC, if data were absent for HIV clinic visits and for ED visits, it was assumed that missing = no visit. For other variables, especially if data were available from more than one source, e.g. housing (case management notes and Ryan White Services report), there were relatively few missing datapoints. However, for other variables such as income and insurance, which were only collected in the outpatient clinic setting (not in jail), there were a lot of missing data (up to 29%) which were not missing at random, therefore missing was treated as a separate category for these variables. All statistical analyses were run using SAS, version 9.4 (SAS Institute Inc., Cary, NC).

## Results

Of 2473 incarcerations of HIV-positive individuals identified during the study period, 669 were excluded for not being released to the community, 163 planned to seek care outside of the Dallas Ryan White-funded system, and 188 were excluded due to having less than 90 days of follow-up in the study period. The final study population consisted of 1450 incarcerations involving 1155 unique individuals.

The general characteristics of the study population are shown in Table 1. The majority of incarcerations were among Blacks (68%), the mean age was 39.1 years, and 77% were male. Only about one quarter reported a stable/permanent living situation, and employment was 8.3% overall. Reported use of any illicit drug was 64%, with the most prevalent drug being cannabis (42%). Over half of the cohort reported a history of mental illness (50.1%). In terms of prior HIV care, 63% reported prior ART prescriptions, but only 40% of the overall cohort reported that they had been adherent. Overall, 43% of the cohort did not present to an HIV clinic during the year after release to the community with 34% linked to HIV care within 90 days of jail release, and an additional 23% linked to HIV care after 90 days from release to the community.

**Table 1:** Baseline characteristics of all releases of HIV-positive individuals released from Dallas County Jail to the community, January 2011–November 2013

Demographics and incarceration	No ED visits ***n*** = 685 (%)	1 to 2 ED visits ***n*** = 463 (%)	> 3 ED visits ***n*** = 302 (%)	Total ***N*** = 1450 (%)
Age (mean ± SD)	39.1 ± 10.6	38.2 ± 10.6	40.4 ± 11	39.1 (30.2–47.7)
Male (%)	81.0	76.5	67.9	76.8
**Race/ethnicity**				
White	18.5	17.1	22.9	19.0
Black	64.7	69.3	70.9	67.4
Hispanic	16.8	13.6	6.3	13.6
**Median incarceration length [IQR] (days)**	5 [1–18]	6 [1–18]	7 [2–23]	6 [1–19]
**HIV risk factor**				
IDU + MSM	2.5	4.3	3.0	3.2
IDU (non-MSM)	5.6	7.6	11.6	7.5
MSM (non-IDU)	31.7	30.7	25.5	30.1
All others	60.3	57.5	59.9	59.3
**SES/housing**				
Ever married	19.9	23.1	30.8	23.2
Family aware of HIV status	47.5	55.3	62.3	53.0
Family supportive of HIV status	57.5	60.5	61.6	59.3
Stable/permanent housing	27.7	26.1	21.9	26.0
**Source of Income**				
Employed	9.6	8.2	5.3	8.3
Disability benefits	19.9	30.2	39.7	27.3
All others	70.5	61.6	55.0	64.4
**History of physical/sexual abuse**	21.2	23.8	32.5	24.3
**Income level**				
Percent at or below 100% FPL	64.7	72.6	74.5	69.2
Percent above 100% FPL	6.1	5.6	3.3	5.4
Unknown or missing	29.2	21.8	22.2	25.4
**Insurance coverage**				
Commercial	2.3	1.7	3.0	2.3
Medicare	10.7	13.4	13.6	12.1
Medicaid	16.8	30.0	44.7	26.8
Charity	42.5	37.8	27.2	37.8
Missing	27.7	17.0	11.5	21.0
**Substance use**				
At-risk alcohol use	4.8	5.6	6.6	5.5
Tobacco use	48.8	58.1	67.2	55.6
Any illicit drug use	58.0	66.7	67.2	62.8
Cannabis	39.9	46.7	40.7	42.2
Cocaine	24.2	24.6	34.4	26.5
Crack cocaine	15.3	16.4	26.8	18.1
Methamphetamines	12.4	13.6	15.2	13.4
Heroin	5.0	7.3	12.2	7.2
Stimulant/opioid use	40.6	41.3	54.0	43.6
**Mental illness**				
Any mental illness	43.4	52.7	61.3	50.1
Bipolar disorder	23.8	30.0	36.1	28.3
Schizophrenia/psychosis	13.6	18.8	21.2	16.8
Anxiety	8.5	8.4	11.3	9.0
Depression	26.0	34.3	38.4	31.2
PTSD	2.0	3.2	6.6	3.4
Serious mental illness	28.6	34.8	42.7	33.5
**HIV care**				
Reported prior HIV clinic	56.9	64.2	61.6	60.2
Prescribed ART before incarceration	62.3	63.1	64.6	63.0
Adherent to ART before incarceration	42.3	39.7	38.4	40.7
First viral load in jail undetectable	35.7	28.4	27.7	31.6
Last viral load in jail undetectable	38.4	31.6	34.2	35.3
**Linkage to care**				
No clinic visit after release	46.3	39.3	40.1	42.8
First clinic visit 0–90 days after release	33.1	36.3	30.8	33.7
First clinic visit > 90 days after release	20.6	24.4	29.1	23.6

### Outcomes

**Fig. 1:**
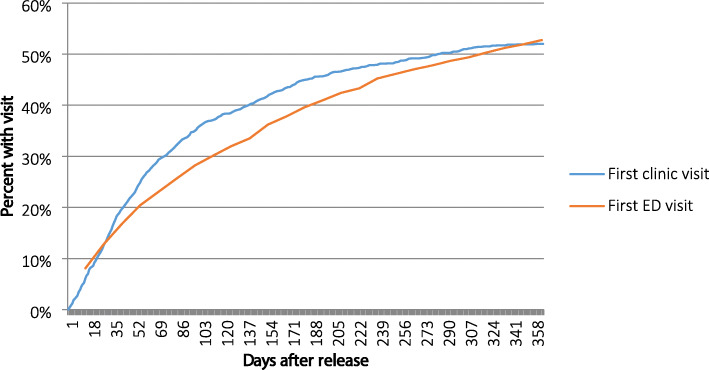
Percentage with first HIV clinic visit and first ED visit after jail release. HIV=Human Immunodeficiency Virus, ED = Emergency Department

The overall proportion of the study population with at least one ED visit after jail release was 53% (Fig. 1). Among the 765 HIV-positive jail released detainees that presented to the ED, 463 (60.5%) had 1 to 2 ED visits in the follow up period, whereas 302 (39.5%) had 3 or more ED visits. In the univariate analysis, the major predictors of ED utilization were female gender, being married, family awareness of HIV status, receiving disability benefits, prior physical and sexual abuse, mental illness, substance use, prior HIV care before incarceration, and late engagement to HIV care after jail release. Hispanics were significantly less likely to present to the ED than whites (Table 2).

**Table 2:** Univariate and multivariate predictors of ED utilization

Independent variable	Univariate analysis		Multivariate analysis	
	Unadjusted OR (95% CI)	***p*** value	Adjusted OR (95% CI)	***p*** value
**Demographics and incarceration**				
**Race/ethnicity**				
White	(Reference)	–	(Reference)	–
Black	0.96 (0.75–1.24)	0.76	1.04 (0.80–1.35)	0.78
Hispanic	0.53 (0.37–0.76)	0.0004	**0.61 (0.42–0.87)**	**0.007**
**Age (per 5 years in units)**	1.02 (0.98–1.07)	0.30	–	–
**Female gender (vs. male)**	1.66 (1.32–2.08)	< 0.0001	**1.30 (1.00–1.69)**	**0.05**
**Days of incarceration**	1.002 (1.000–1.004)	0.10	–	–
**HIV risk factor**				
Heterosexual	(Reference)	–	–	–
MSM (non-IDU)	0.90 (0.72–1.11)	0.32	0.73 (0.51–1.06)	0.25
IDU	1.60 (1.17–2.20)	0.004	0.73 (0.51–1.04)	0.21
**SES/housing**				
Ever married	1.51 (1.20–1.89)	0.0004	1.25 (0.98–1.60)	0.08
Family aware of HIV status	1.56 (1.28–1.89)	< 0.0001	**1.32 (1.06–1.65)**	**0.02**
Family supportive of HIV status	1.14 (0.94–1.39)	0.70	–	–
Stable housing	0.82 (0.65–1.02)	0.07	–	–
**Source of income**				
Employed (vs. other)	0.84 (0.58–1.21)	0.35	–	–
Disability benefits (vs. other)	2.04 (1.64–2.54)	< 0.0001	–	–
h/o physical/sexual abuse	1.49 (1.19–1.86)	0.0005	1.20 (0.94–1.54)	0.15
**Substance use**				
At-risk alcohol use	1.28 (0.84–1.95)	0.25	–	–
Tobacco use	1.74 (1.43–2.12)	< 0.0001	–	–
Any illicit drug use	1.42 (1.16–1.74)	0.0007	–	–
Cannabis	1.11 (0.91–1.35)	0.30	–	–
Club drug	1.14 (0.78–1.66)	0.50	–	–
Cocaine	1.37 (1.10–1.70)	0.005	–	–
Crack cocaine	1.62 (1.26–2.07)	0.0001	–	–
Heroin	2.09 (1.45–3.01)	< 0.0001	–	–
Opiates	1.85 (0.56–6.17)	0.32	–	–
Methamphetamines	1.19 (0.90–1.57)	0.23	–	–
Prescription medications	1.19 (0.49–2.90)	0.70	–	–
Stimulant/opioid use	1.39 (1.14–1.68)	0.001	1.07 (0.86–1.34)	0.06
**Mental illness**				
Any mental illness	1.70 (1.40–2.07)	< 0.0001	–	–
Bipolar	1.55 (1.26–1.92)	< 0.0001	–	–
Schizophrenia/psychosis	1.52 (1.18–1.96)	0.001	–	–
Anxiety	1.22 (0.87–1.70)	0.24	–	–
Depression	1.56 (1.27–1.92)	< 0.0001	–	–
PTSD	2.54 (1.50–4.31)	0.0005	–	–
Serious mental illness	1.57 (1.28–1.92)	< 0.0001	**1.28 (1.02–1.61)**	**0.03**
**Prior HIV care**				
Prior HIV clinic before incarceration	1.23 (1.01–1.50)	0.043	–	–
Prescribed ART before incarceration	1.07 (0.87–1.30)	0.53	–	–
Adherent to ART before incarceration	0.88 (0.72–1.07)	0.21	**0.77 (0.62–0.95)**	**0.01**
ART prescribed in jail	0.87 (0.71–1.07)	0.19	–	–
**Linkage to care**				
Clinic visit 0–90 days after release	(Reference)		(Reference)	
Clinic visit > 90 days after release	1.31 (1.01–1.69)	0.0018	**1.33 (1.02–1.73)**	**0.04**
No clinic visit after release	1.13 (0.90–1.42)	0.35	0.80 (0.63–1.01)	0.06

*Abbreviations*: *ED* Emergency Department, *CI* Confidence Interval, *HIV* Human Immunodeficiency Virus, *MSM* Men who have sex with men, *IDU* Injection drug use, *PTSD* Post-traumatic stress disorder, *ART* Anti-retroviral therapy

In multivariate logistic regression analysis, female patients were significantly more likely to present to the ED than male patients (aOR 1.30 [95% CI: 1.00–1.69]). Family awareness of HIV status, severe mental illness, and late engagement to HIV care after jail release were all significantly associated with higher ED utilization. Conversely, Hispanic ethnicity and adherence to ART prior to incarceration were significant predictors of less ED utilization in the multivariate analysis (Table 2).

**Fig. 2:**
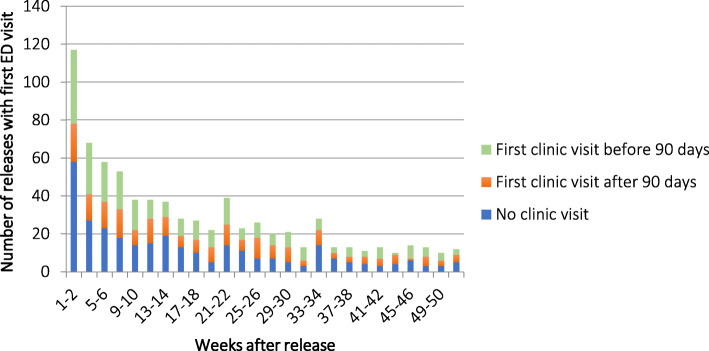
Timing of first ED visit by HIV care engagement group. HIV=Human Immunodeficiency Virus, ED = Emergency Department

ED utilization was high shortly after transitioning to the community with 27% of the entire cohort having at least one ED visit by 90 days after release. Furthermore, there was higher percentage of first ED visits than first clinic visits during the first 30 days after jail release (Fig. 1 enlargement). The majority of first ED visits were from patients that had delayed linkage to HIV care (> 90 days post-release) or had no linkage to HIV care whatsoever (Fig. 2).

**Fig. 3:**
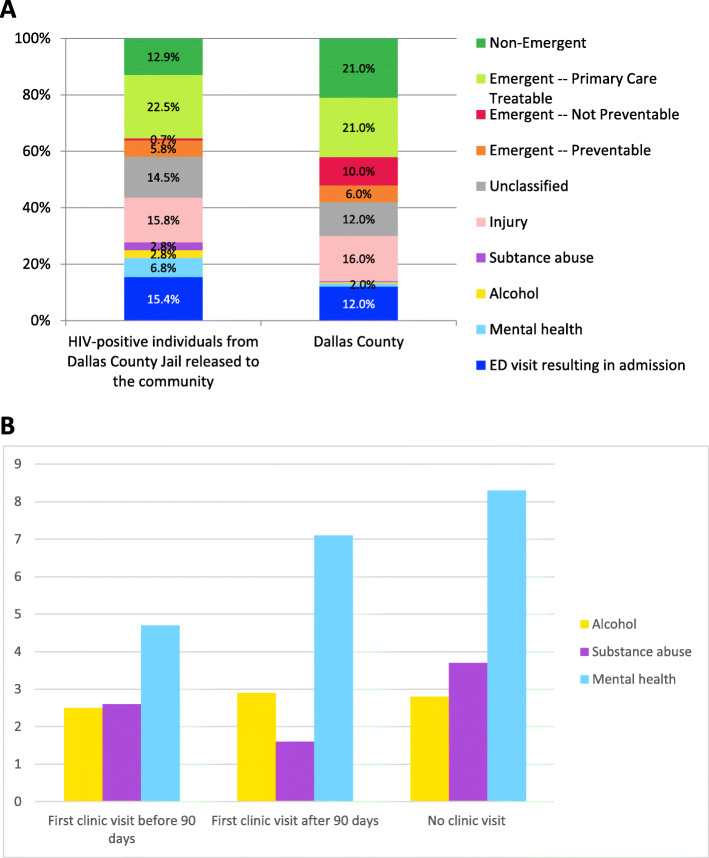
**a** NYU ED visit categorization, HIV-positive released jail detainees versus general Dallas county population. Dallas County data adapted from Sharma S, et al. High frequency patient analysis to identify disparities associated with emergency department utilization in Dallas County. Texas Public Health Journal. 2017;69:19–29; NYU=New York University, ED = Emergency Department, HIV=Human Immunodeficiency Virus. **b** NYU ED categorization for alcohol, substance abuse and mental health by HIV care engagement group

Our cohort had a total of 2771 ED visits in the 12 months following release from jail. Only 15.4% ED visits resulted in hospitalizations. Using the NYU ED classification algorithm, 29% of ED visits were emergent, 12.9% were non-emergent, 14.5% were unclassified and the remainder were either related to injury (15.8%), mental health (6.8%), alcohol use (2.8%), or substance use (2.8%) (Fig. 3a). Compared to the general population of Dallas County, our study population lower ‘emergent-not preventable’ causes for their ED visits and a higher proportion of ED visits related to substance use, alcohol or mental health (Fig. 3b). The NYU ED classification was compared within the three different linkage to HIV care groups (early, late or no linkage to HIV care) obtaining similar percentages (data not shown).

## Discussion

Early and frequent emergent department utilization occurred among PLWH recently released from a large urban jail in the South. In our cohort, an initial visit to the ED visit was more common than first attending an HIV clinic visit during the first month after jail release. Almost one-quarter of the jail releasees visited the ED at least once during the first 90 days following community reentry and over half of the entire cohort had at least one post-release ED visit within a year of release. Among the individuals who visited the ED, four out of ten had three or more ED encounters. These numbers reflect the substantial burden of high ED utilization immediately following jail release and raises the question of whether some visits could be prevented.

Compared to other groups released from incarceration, PLWH had similarly high rates of ED utilization (Frank et al., 2013), despite that PLWH typically receive care in a patient centered medical home (Friedman, Crowley, Howard, & Pavel, 2015). Compared to the general population of Dallas County (Sharma et al., 2017), ED visits related to substance use, alcohol or mental health were 5 times more common among PLWH released from jail than the general population. PLWH recently released from jail also had a higher proportion of ED visits resulting in hospital admission than the general population.

Our study confirms and extends the literature by incorporating regional ED data which allows us to more comprehensively estimate the burden of ED utilization by PLWH after jail release. In accordance with previous studies (Boyd et al., 2015; Frank et al., 2013; Khanna et al., 2019; Meyer et al., 2013), individuals released from incarceration who engaged late into HIV care (first HIV clinic visit after 90 days from jail release) visited the ED earlier and more frequently than the ‘early linkers’ (first HIV clinic visit before 90 days from jail release). Furthermore, late linkage to HIV care was an independent predictor of high ED utilization in our multivariate analysis after adjusting for demographic characteristics, socio-economic status, mental health, substance use and prior engagement in HIV care. Our results highlight similar patterns of health care utilization compared to the northeastern US and Canada, but on a greater scale, incorporating data from a large metropolitan area in the Southern United States, a region disproportionally affected by the HIV/AIDS epidemic with fewer social services and overall worse HIV outcomes.

In our study, we found that mental health and substance use disorder disproportionally impact PLWH after jail release, consistent with the existing literature (Boyd et al., 2015; Frank et al., 2013; Khanna et al., 2019; Meyer et al., 2013). According to the NYU ED visit severity algorithm, 10% of all ED visits in our cohort were related to mental health and substance use disorder. Additionally, serious mental illness was a statistically significant predictor of high ED utilization in our multivariate analysis. In other comparisons of ED utilization between ex-prisoners and the general population, Frank et al. demonstrated that people released from prison in Rhode Island were more likely to have an ED visit for substance use and psychiatric comorbidities, when compared to the general population (Frank et al., 2013). In particular, PLWH who have mental health and/or substance use disorders have been identified as high utilizers of ED services in the period immediately following release from incarceration. Boyd et al. evaluated a cohort of 71 PLWH in the 12 months following jail release and demonstrated that substance use not only accounted for a high percentage (31%) of ED visits, but was also associated with early and frequent ED utilization after release (Boyd et al., 2015). In a study by Meyer et al., the cumulative burden of psychiatric illness was also positively correlated with frequent ED visits by PLWH released from prison (Meyer et al., 2013). Our study goes beyond the existing data to demonstrate that released PLWH who present late (> 90 days) to HIV clinic or not at all, experience an increasing proportion of ED visits related to mental health issues.

Incarcerated women constitute another vulnerable group in the correctional system. They represent the fastest growing population in the criminal justice system and account for an increasing proportion of newly diagnosed HIV cases (Maruschak, 2015; Subramanian, Henrichson, & Kang-Brown, 2015). Women living with HIV who are incarcerated are also more likely to suffer from substance use, depression and to be victims of past physical and sexual abuse (Mignon, 2016). Criminal justice-involved women are also more likely to be frequent ED utilizers than males (Meyer et al., 2013). Health services for incarcerated women are often not gender-specific nor individualized to meet the particular psychosocial needs of women. Trauma-informed care (TIC) has emerged as an approach to healthcare that incorporates safety, trust, peer support, collaboration, empowerment, and cultural perspectives to improve quality of care and the patient experience, thereby increasing longitudinal engagement of marginalized and hard-to-reach patients, such as those with criminal justice system exposure (Chaudhri, Zweig, Hebbar, Angell, & Vasan, 2019). Implementation of TIC in the vulnerable and high-risk HIV jail population might improve healthcare quality and engagement into HIV care after correctional release and at time of ED evaluation.

Other independent predictors of ED utilization in our multivariate model were family being aware of HIV status (higher ED visits) and Hispanic ethnicity (lower ED visits). It is possible that PLWH who have disclosed their HIV status to their relatives have stronger family support which is a facilitator of health care access (Bracken, Hilliard, McCuller, & Harawa, 2015). The association between Hispanic ethnicity and fewer ED visits may be related to higher rates of post-release linkage to HIV care among Hispanics in our study, as we have discussed in our previous publication, and correlates with lower proportions with mental illness and substance use among Hispanics in our study (Ammon et al., 2018).

Several interventions focused on improving retention in routine HIV care after incarceration have been evaluated. Transitional case management programs and peer navigation have been shown to help PLWH engage in health care after jail release and improve HIV treatment outcomes (Althoff et al., 2013; Sayles, Wong, Kinsler, Martins, & Cunningham, 2009; Spaulding et al., 2013). However, conflicting data exist, and some studies have shown no benefit of intensive management interventions over control groups on post-release outcomes, though these programs had very high linkage to care in the control arm and high recidivism rates, making post-release outcomes more challenging to interpret (MacGowan et al., 2015; Wohl et al., 2011, 2017). The expansion of Medicaid is an important tool for improving care delivery for individuals leaving jail or prison (Guyer, Serafi, Bachrach, & Gould, 2019), though individuals released in states which have not expanded Medicaid, particularly low-income individuals, face additional barriers (Grodensky et al., 2018; Washington Post Editorial Board, 2019). Ending the Medicaid exclusion for people under correctional supervision would allow for continuous access to mental health and substance abuse treatment which is associated with lower rates of recidivism (Pew Charitable Trusts, 2011) and could impact healthcare utilization. However, our findings indicate that half of the releasees with the highest number of ED visits (3 or more) were Medicaid beneficiaries which may indicate that Medicaid facilitates health care access but does not necessarily optimize health care utilization. Future interventions should also focus on ED encounters as opportunities to reengage PLWH into HIV care. As demonstrated by a study of a routine opt-out HIV testing program in a large Houston emergency room, care coordination efforts initiated in the emergency room improve engagement, retention, and virologic suppression in previously diagnosed patients (Flash et al., 2015). Lastly, effective reforms to address the mass incarceration problem in the US would also have a significant impact on the burden of ED misuse after prison or jail release by reducing the exposure to the destabilizing post-release period (Dumont, Brockmann, Dickman, Alexander, & Rich, 2012).

Despite the number of strengths of this research project, our study has several limitations. First, our study involves data collected in a single county jail which may not be generalizable to other settings, although we found similarities between our results and other cohorts of PLWH released from correctional supervision. Second, it is possible that some of the HIV-positive released jail detainees received HIV care outside the Ryan White care system in the Dallas metropolitan area (although we excluded patients who mentioned that they planned to do so), potentially resulting in decreased observed rates of linkage to HIV care in this study. Lastly, there is a sizeable proportion of patients with ED visits unclassified by the NYU ED visit severity algorithm (14.5%) which were not further characterized. This is similar to other studies using this algorithm and a limitation to using this approach (Johnston, Allen, Melanson, & Pitts, 2017).

## Conclusions

In this study using a comprehensive database of hospitals, jail health data and data from community-based HIV clinics in North Texas, we demonstrated that high ED utilization by PLWH occurs shortly and frequently after jail release. In this population, disproportionally impacted by the HIV/AIDS epidemic in the United States, we identified a higher proportion of ED encounters for mental health and substance use disorders than the general population. Similarly, we demonstrated that women, persons with serious mental illness and late engagers in HIV-care are high utilizers of emergent department resources. Interventions to improve post-release linkage to HIV care are key to decrease the burden of ED misuse and should be focused on vulnerable populations such as women, patients with severe mental health and substance use disorder. These actions are important in the efforts to end the HIV/AIDS epidemic.

## Data Availability

The datasets generated and analysed during the current study are not publicly available due the fact that the data come from multiple sources and are covered under multiple different data use agreements but are available from the corresponding author on reasonable request.

## Abbreviations

PLWH: People living with HIV
ED: Emergency department
HIV: Human immunodeficiency virus
AIDS: Acquired Immune Deficiency Syndrome
ART: Antiretroviral therapy
NYU: New York University
DCJ: Dallas County Jail
PHHS: Parkland Health and Hospital System
RSR: Ryan White Service Report
eUCI: Encrypted unique client identifier
IDU: Injection drug use
MSM: Men who have sex with men
EMR: Electronic medical record
FPL: Federal poverty level
DFWHC: Dallas Fort Worth Hospital Council
US: United States
TIC: Trauma-informed care
